# Primary Pericardial Synovial Sarcoma: A Case Series and Review of Literature

**DOI:** 10.7759/cureus.86731

**Published:** 2025-06-25

**Authors:** Harmanjit Kaur, Tushar Kumar, Surbhi Singh, Lakshmisree A Vemulakonda, Rajat Bhargava

**Affiliations:** 1 Department of Hematology and Oncology, Boston Medical Center, Boston, USA; 2 Department of Radiology, University of Washington School of Medicine, Seattle, USA; 3 Department of Hematology and Oncology, The Brooklyn Hospital Center, Brooklyn, USA; 4 Department of Pathology, University of Washington School of Medicine, Seattle, USA

**Keywords:** cardiac tumors, pericardial sarcoma, primary pericardial sarcoma, spindle cell sarcoma, thoracic oncology

## Abstract

Primary pericardial synovial sarcoma (PPSS) is a rare and aggressive cancer that arises from pluripotent mesenchymal cells of the pericardium. The pathognomonic genetic hallmark is the chromosomal translocation t(X;18)(p11;q11), resulting in the SS18-SSX fusion oncogene, which, down the line, disrupts transcription regulation and chromatin remodeling, ultimately leading to carcinogenesis. In our article, we describe two cases of PPSS in previously healthy young men, managed with multidisciplinary teams, along with a review of the literature of cases reported to date. Both of our patients are young adults, with very different presentations in terms of symptoms, one presenting with shortness of breath (SOB) and the second with chest pain. Both patients had imaging studies that reported a pericardial mass. Ultimately, a diagnostic evaluation was done, with fluorescence in situ hybridization (FISH) confirming primary pericardial synovial sarcoma. Unfortunately, both patients passed away within a week of diagnosis.

A comprehensive search of public databases, including PubMed and Google Scholar, was conducted up to 2025. The search criteria included synovial pericardial sarcoma, sarcoma of the heart, cardiac sarcoma, and primary pericardial synovial sarcoma. The search yielded over 100 results, of which 46 articles focused specifically on primary pericardial synovial sarcoma. This article summarizes all reported PPSS cases to date, including patient age, race, gender, symptoms, cytological analysis, and histological subtypes.

## Introduction

Primary pericardial synovial sarcoma (PPSS) is an extremely uncommon malignant tumor of the pericardium. It belongs to a broader category called synovial sarcomas (SS), which is a rare type of sarcoma accounting for 5%-10% of all soft tissue sarcomas and is most commonly seen in young adults aged 15-40 years, with a slight male predominance [1]. SS often occurs in the extremities near joints [2], although it can rarely appear in other sites such as the thoracic cavity. PPSS is incredibly rare, with only 46 documented cases in the literature till 2025. It was first described by McAllister and Fenoglio in 1978 in a publication named "Tumors of the cardiovascular system" [3]. Given its rarity, the precise incidence of PPSS remains unclear. The t(X;18)(p11.2;q11.2) translocation, which results in the SS18-SSX fusion gene and induces cancer development, is the hallmark feature of synovial sarcoma [4]. The diagnosis of PPSS is particularly challenging given its nonspecific presentation, which includes dyspnea, chest discomfort, and evidence of pericardial effusion. This tumor has a mesenchymal origin and histological similarities to soft tissue synovial sarcoma. Histologically, PPSS may be monophasic, characterized by spindle cells (SS), or biphasic, containing both spindle cells and epithelial components. PPSS expresses tumor markers such as transducin-like enhancer of split-1 (TLE1) and B-cell lymphoma 2 (Bcl-2), which can differentiate PPSS from other sarcomas, highlighting the importance of immunohistochemical (IHC) investigations as an important tool to rule out other sarcomas. Advanced radiological studies such as cardiac magnetic resonance imaging (MRI) and contrast-enhanced computed tomography (CT) scans are often utilized as they help identify tumor size and local spreading, guiding the approach for tumor resection. Early diagnosis can help prevent local spread, hence improving the outcome of treatment; however, it has a high recurrence rate, with an average of less than a year. Despite the wide range of available treatments, the general prognosis remains poor, with median survival rates often falling below two years.

We add two cases to the already existing 46 reported cases of PPSS. These two cases illustrate presentations of an aggressive malignancy in young, otherwise healthy patients with no family history of cancer, emphasizing both the diagnostic and management challenges associated with this rare entity. The first patient presented with weeks of unexplained shortness of breath (SOB), a vague symptom that ultimately revealed a massive pericardial tumor with extensive invasion up to the renal arteries. Despite requiring seven cycles of chemotherapy just to make surgical resection feasible, his postoperative course was marked by rapid deterioration, including acute respiratory distress syndrome (ARDS) and cardiogenic shock, ultimately leading to comfort-focused care. In contrast, the second patient presented with chest pain suggestive of acute coronary syndrome. On imaging, he was found to have tumor invasion more locally into the left circumflex artery and ventricles. He suffered a fatal myocardial infarction shortly after the biopsy, highlighting the diagnostic challenges during diagnosis, given its proximity to vital structures.

## Case presentation

Case 1

A 23-year-old male patient with a history of well-controlled childhood asthma presented to the emergency department with worsening shortness of breath for two months. His prior history was also notable for vaping and marijuana use. His family history was notable for mitral valve prolapse in his maternal aunt. Vitals were normal at presentation with blood pressure of 140/80 mmHg and trace SpO2 of 98%. On examination, accessory muscle usage for aspiration was noted, and mild wheezing was present. Heart sounds were normal with no murmur heard. Laboratory investigations revealed a B-type natriuretic peptide (BNP) of 450 pg/mL, raising a small suspicion of heart failure. Other laboratory parameters were unremarkable.

**Figure 1:**
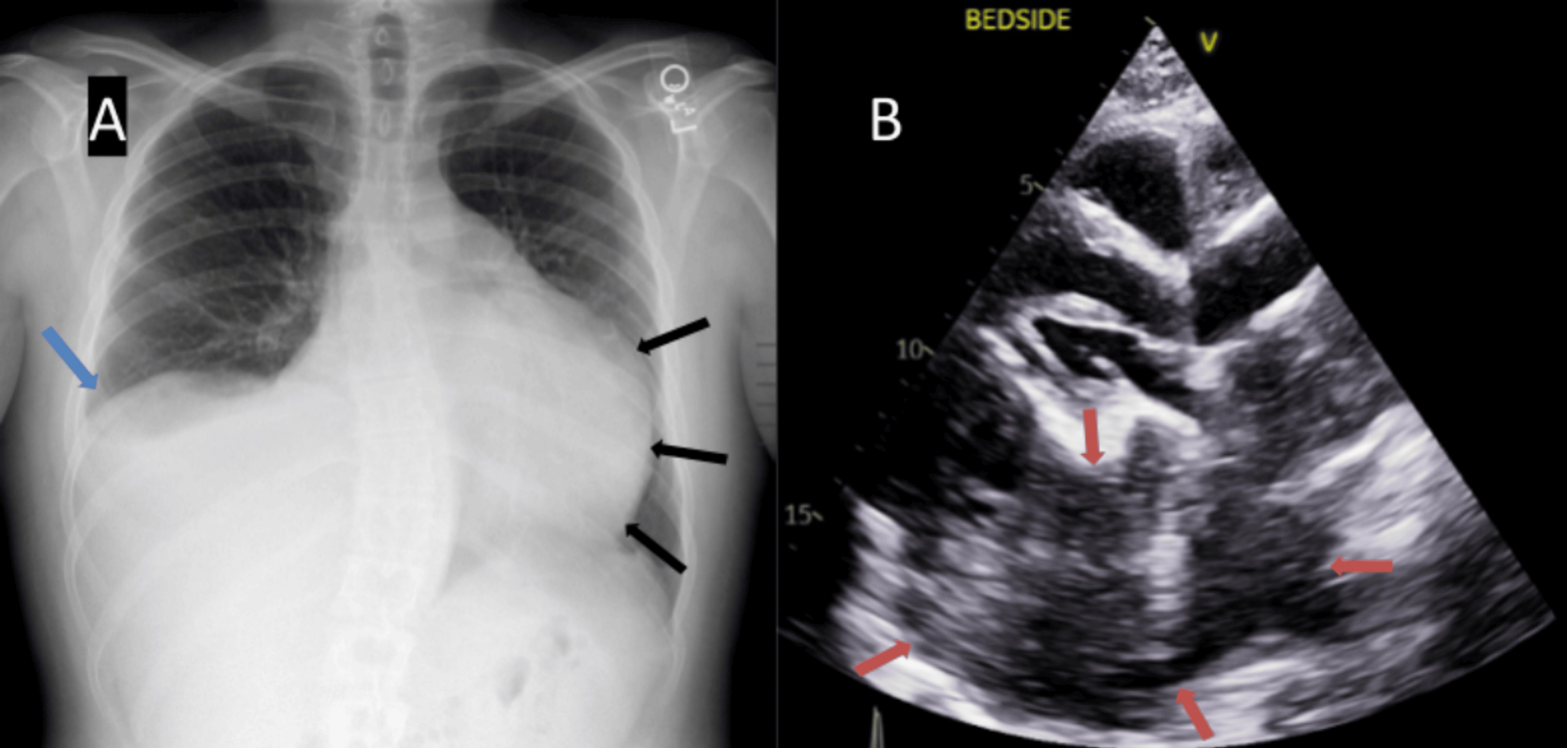
(A) Radiograph of the chest (PA view) with cardiomegaly (black arrows in A) and right small pleural effusion (blue arrow in A). (B) Bedside echocardiogram with large posterior pericardial mass (orange arrows in B). PA: posteroanterior

A radiograph of the chest revealed cardiomegaly and a small right pleural effusion. A bedside echocardiography was abnormal and demonstrated an intermediate left ventricular ejection fraction of 45%. An echo density was seen surrounding the heart, predominantly along the posterior wall, with constrictive filling of inflow. There were external constraints on diastolic expansion along the posterior and lateral walls of the heart due to a large pericardial mass, which was also seen to compress the left atrium. The right ventricular size was small, with severe systolic dysfunction. There was possible external compression of the pulmonary artery and possible supravalvular pulmonary valve stenosis (peak 2.6 ms gradient 10 mmHg). Small pericardial effusion was also seen with no superior vena cava (SVC) syndrome (Figure 1).

**Figure 2:**
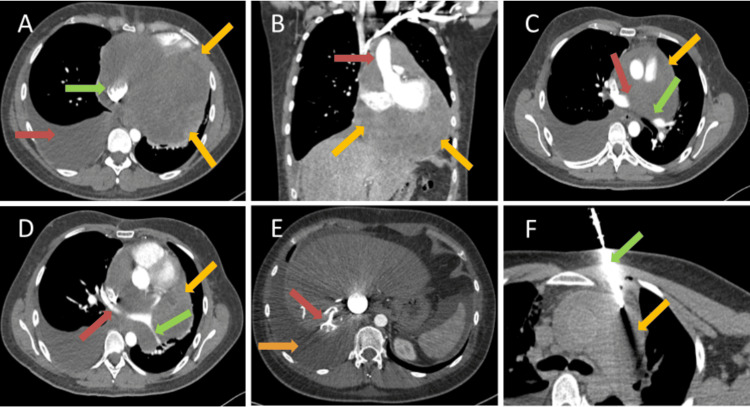
(A) Axial CECT of the lower chest demonstrating a large pericardial mass (yellow arrows in A), encasing the IVC (green arrow in A), with moderate-sized right pleural effusion (red arrow in A). (B) Coronal CECT of the chest demonstrating a large pericardial mass (yellow arrows in B), encasing the ascending aorta (red arrow in B). (C) Axial CECT of the upper chest demonstrating a pericardial mass (yellow arrow in C), encasing the right (red arrow in C) and left (green arrow in C) pulmonary arteries. (D) Axial CECT of the mid chest demonstrating a large pericardial mass (yellow arrow in D), encasing the right inferior (red arrow in D) and left inferior (green arrow in D) pulmonary veins. (E) Axial CECT of the upper abdomen demonstrating reflux of contrast into the hepatic veins (red arrow in E) and right pleural effusion (orange arrow in E). (F) CT-guided biopsy (green arrow in F) of pericardial mass (yellow arrow in F). CECT: contrast-enhanced computed tomography, IVC: inferior vena cava, CT: computed tomography

The initial differential considerations for this mass were either a lymphoma or a pericardial metastasis. Right heart catheterization and strain imaging were not performed as per the primary team's discretion, as the patient had significant orthopnea. A chest CT was performed to evaluate the pericardial mass (Figure 2).

A large hypoenhancing heterogeneous mass was seen involving the posteroinferior pericardium and encasing the left superior and inferior pulmonary veins, bilateral renal arteries, main pulmonary artery trunk, and ascending aorta. It was also seen to cause significant compression over the bilateral atria and left ventricle. Reflux of contrast into the hepatic veins was noted as a result of right heart pressure overload. Moderate-sized right pleural effusion was aspirated. The pleural fluid was transudative, and cytology was negative for malignancy. The patient was admitted, and a CT-guided biopsy of the pericardial mass was performed.

**Figure 3:**
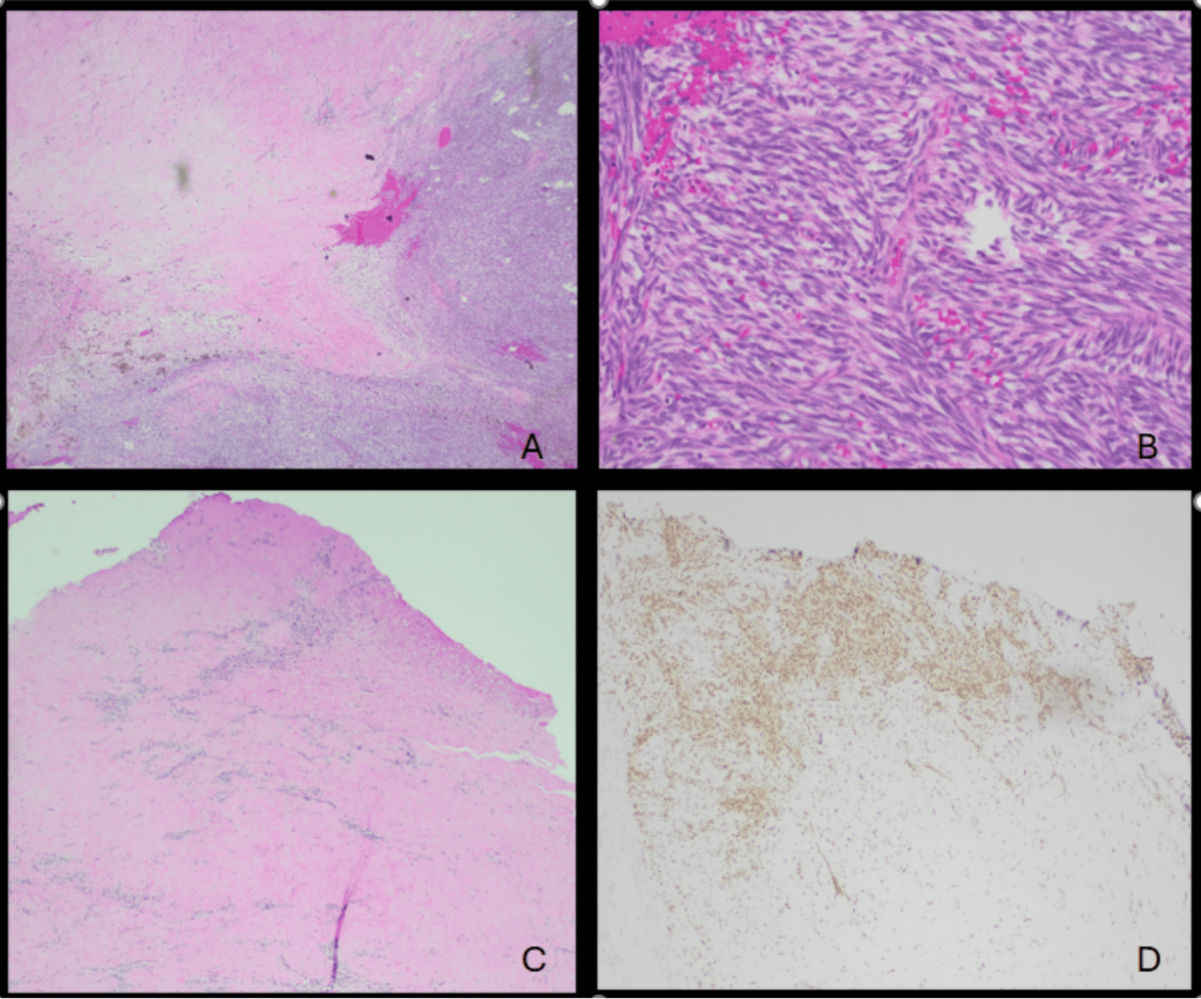
(A) H&E stain of a representative section of the pericardial tumor at 20× magnification showing viable tumor, hyalinization, and hemosiderin-laden macrophages, consistent with treatment effect. (B) H&E stain of a representative section of the pericardial tumor at 200× magnification showing delicate, uniform, and relatively small spindle cells, arranged in dense cellular sheets or vague fascicles, with sparse cytoplasm and ovoid, hyperchromatic nuclei with regular granular chromatin, high N:C ratio, and inconspicuous nucleoli. (C) H&E stain of a representative section of the pericardial tumor at 20× magnification showing tumor necrosis, hyalinization, and viable tumor. (D) SS18-SSX immunostain demonstrating positive expression in viable tumor cells. H&E: hematoxylin and eosin

Pathology demonstrated a monotonous, hypercellular neoplasm with small- to indeterminate-sized cells. The nuclei were hyperchromatic with irregular nuclear borders and associated vacuoles (Figure 3).

Five mitoses per 10 high-power fields were identified. Variable myxoid changes were also seen. The neoplastic cells were positive for CD99 and weakly positive for TLE1. Specific scoring or quantification for immunohistochemical (IHC) markers is not available, but IHC stains were negative for CD45, SOX10, SALL4, Pan CK, INSM1, CD3, CD20, STAT6, desmin, SMA, CD34, WT1 (cytoplasmic staining only), p40, TdT, and CD5. These features favored the diagnosis of indeterminate-grade (FNCLCC grade 2 or 3) synovial sarcoma of the pericardium. The tumor was composed of spindle and round cells, with differentials concerning monophasic synovial sarcoma and round cell sarcoma. Lactate dehydrogenase (LDH), human chorionic gonadotropin (hCG), and alpha-fetoprotein (AFP) were normal.

**Figure 4:**
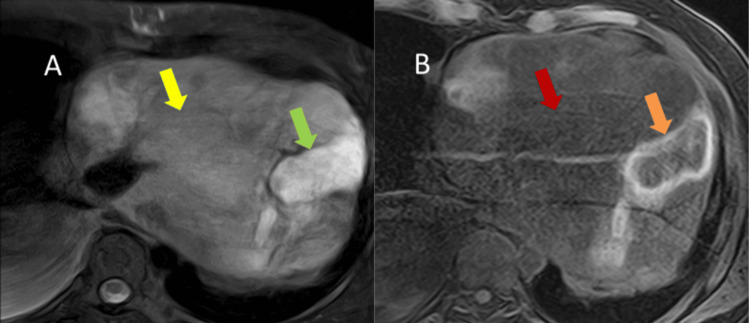
(A) Axial T2W MRI of the lower chest with a large pericardial mass appearing T2 hypointense (yellow arrow), with few areas of moderate T2 hyperintensity (green arrow). (B) Axial T1W post-contrast MRI of the lower chest with hypoenhancing mass (red arrow), with few areas of heterogeneous enhancement (orange arrow). T2W: T2-weighted, MRI: magnetic resonance imaging, T1W: T1-weighted

MRI of the chest was also performed to better evaluate the nature of the pericardial mass (Figure 4).

However, the images were suboptimal due to the patient's breathing artifacts. Post-contrast MR images demonstrated areas of necrosis within the mass.

**Figure 5:**
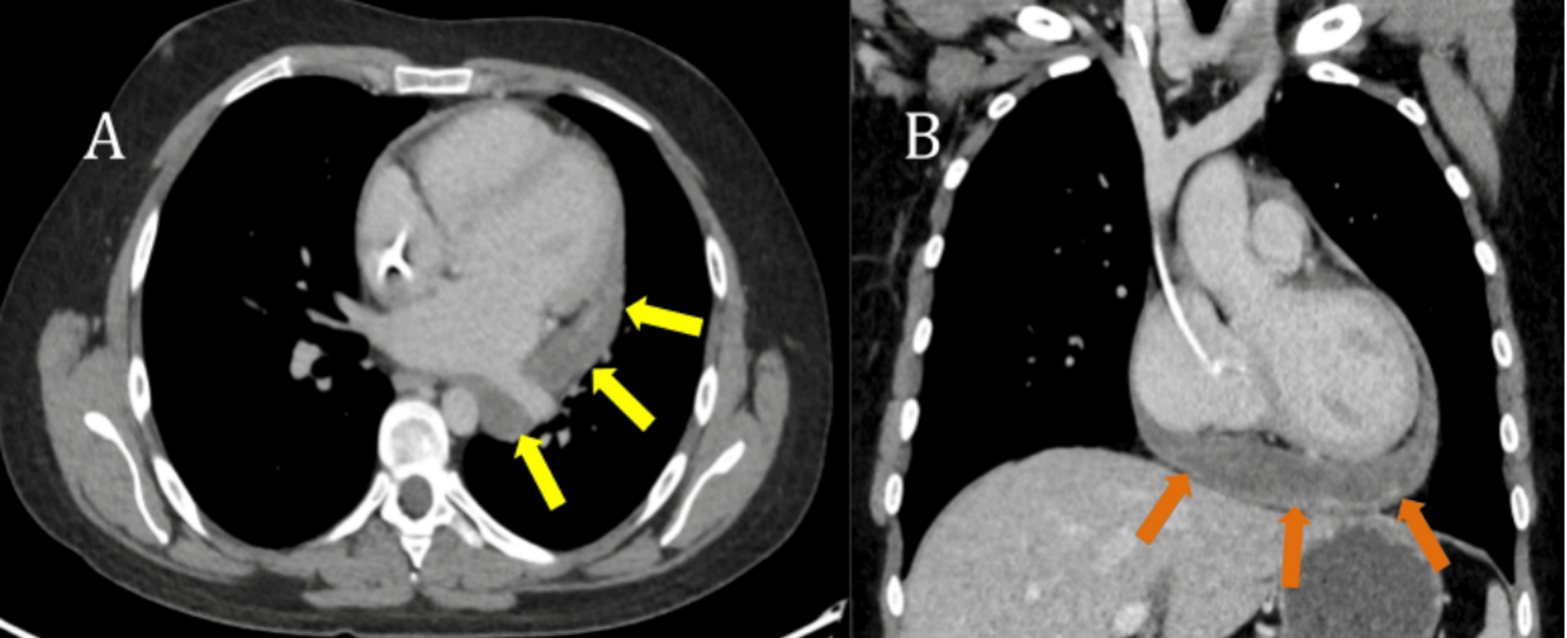
(A) Axial CECT of the chest with a decrease in the size of the pericardial mass (yellow arrows). (B) Coronal CECT of the chest with a decrease in the size of the pericardial mass (orange arrows). CECT: contrast-enhanced computed tomography

Cardiothoracic surgery was consulted; however, they declined resection of the pericardial mass due to its large size. Oncology was consulted, and chemotherapy with ifosfamide with mesna was recommended to decrease the tumor size and make it amenable for surgery. Doxorubicin was withheld due to the patient's low ejection fraction of 43%. After three cycles of ifosfamide plus mesna, for five days each, the patient felt less breathless, and the ejection fraction improved to 47%. Doxorubicin was added to the regimen starting from the fourth cycle as a bolus rather than a 24-hour infusion, with dexrazoxane for cardioprotection. After a total of seven cycles of doxorubicin, ifosfamide, and mesna, a CT scan demonstrated a significant reduction in the size of the pericardial synovial sarcoma with decreased encasement of the major vessels, the maximum thickness of the tumor measuring 2.2 cm at the inferior border, compared to 5.9 cm before the initiation of chemotherapy (Figure 5).

Cardiothoracic surgery was again consulted, and the mass was excised. All gross tumors were successfully removed, and the left atrium was reconstructed with a pericardial patch. Aortic interposition graft was placed, and pericardial reconstruction was done. On postoperative day 3, the patient developed acute respiratory distress syndrome (ARDS) and suffered cardiac arrest. The cardiogenic shock worsened along with severe right ventricular dysfunction. With the consent of the patient's family, life support was withdrawn after seven days when the patient succumbed.

Case 2

**Figure 6:**
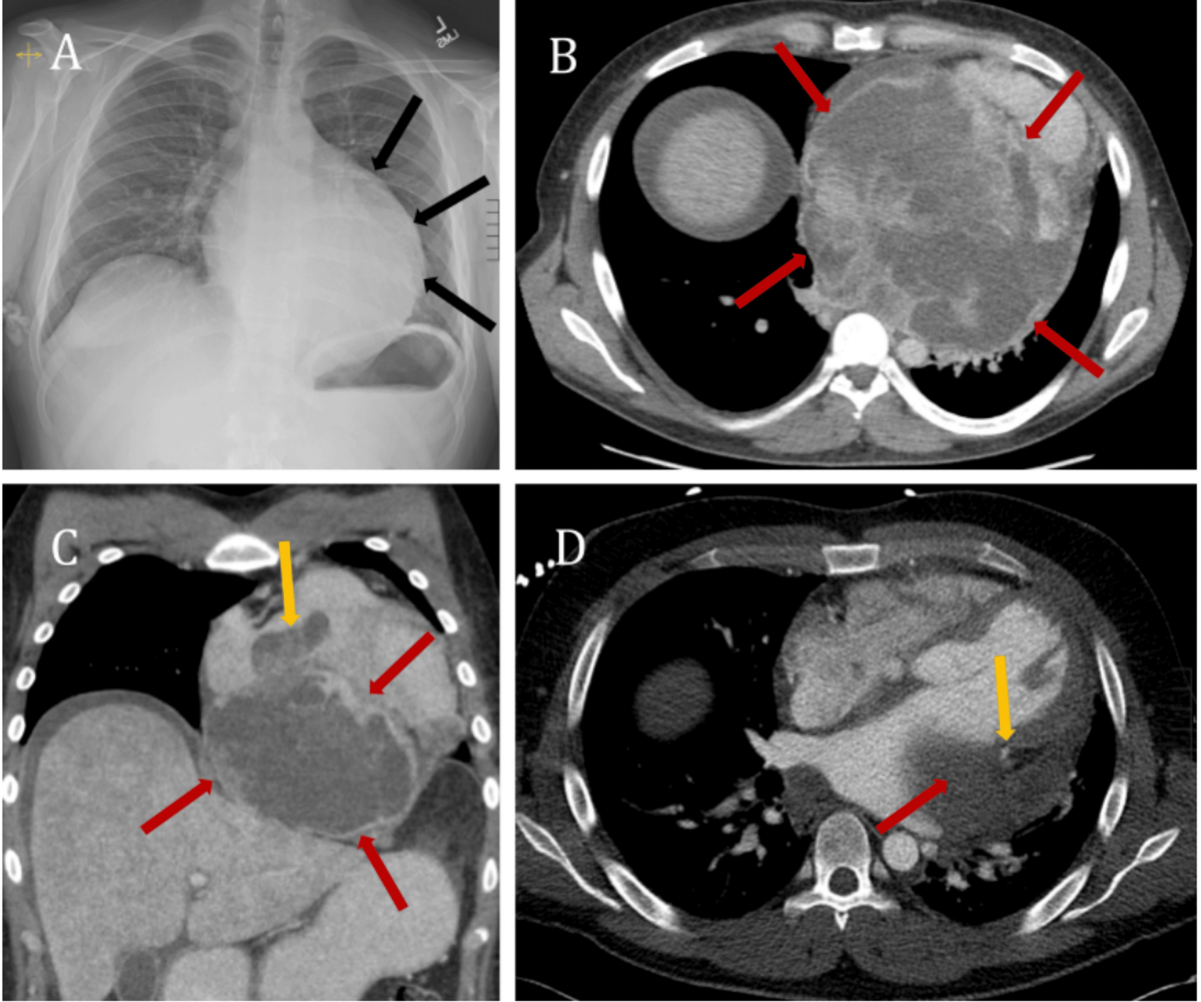
(A) Chest radiograph (PA view) with enlarged cardiac silhouette (black arrows in A). (B) Axial CECT of the lower chest with a large heterogeneously enhancing necrotic mass along the posterior inferior aspect of the heart, originating from the pericardium (red arrows in B). (C) Coronal CECT of the chest with a large pericardial mass (red arrows in C) infiltrating into the right atrium and right ventricle (yellow arrow in C). (D) Axial CECT of the mid chest with the partially visualized pericardial mass (red arrow in D), completely encasing the proximal left circumflex coronary artery (yellow arrow in D). The mid and distal left circumflex coronary artery (not seen in this section) was completely obliterated. PA: posteroanterior, CECT: contrast-enhanced computed tomography

A 31-year-old male patient presented to the emergency department with chest pain. The chest radiograph demonstrated an enlarged cardiac silhouette. An electrocardiogram (ECG) done on admission reported no ischemic changes, and troponins were normal. An echocardiogram was performed, which demonstrated a heterogeneous cardiac mass; however, images are not available. This was followed by a contrast-enhanced cardiac-gated CT scan, which demonstrated a large heterogeneously enhancing partially necrotic mass along the inferior cardiac margin. Infiltration into the right atrium and right ventricle was noted, with complete encasement and obliteration of the distal two-thirds of the left circumflex coronary artery (Figure 6).

**Figure 7:**
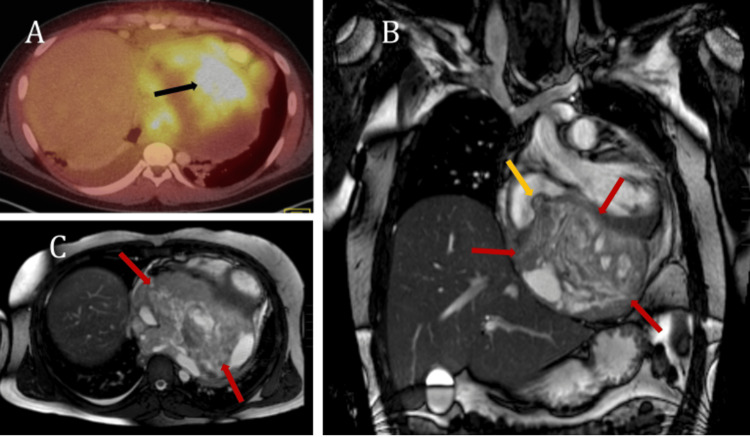
(A) 18F-FDG PET/CT scan with intense tracer uptake in this mass (black arrow in A). (B) Coronal T2W MRI of the chest without contrast with a large heterogeneous pericardial mass (red arrows in B), extending into the right atrium (yellow arrow in B). (C) Axial T2W MRI of the lower chest with heterogeneous pericardial mass (red arrows in C). Significant compromise of cardiac function was noted. 18F-FDG PET/CT: fluorine-18 fluorodeoxyglucose positron emission tomography/computed tomography, T2W: T2-weighted, MRI: magnetic resonance imaging

Positron emission tomography (PET)/CT demonstrated intense tracer uptake along the periphery of the mass. A cardiac MRI was also performed to evaluate cardiac function, which demonstrated decreased end-diastolic volume and the heterogeneous nature of the pericardial mass (Figure 7).

The biopsy and fine needle aspiration cytology slides contained a highly cellular neoplasm containing both spindled and small round blue cell morphologies exhibiting hyperchromasia and scattered mitotic activity. Regions of necrosis were not identified. Immunohistochemical studies revealed that the neoplastic cells were positive for Bcl-2 and CD99, with rare scattered cells also positive for EMA, CAM5.2, pancytokeratin, and desmin. The neoplastic cells lacked expression of SMA, S100, CD31, and CD34. Additionally, cytogenetic studies (Z11-3000) and fluorescence in situ hybridization (FISH) studies (Z11-3194) demonstrated translocation (X;18) in 17 of 18 cells analyzed; also, the Synaptotagmin (SYT) (SS18) gene rearrangement was present in 100% of nuclei examined. The morphologic, immunohistochemical, cytogenetic, and FISH findings supported a diagnosis of high-grade/poorly differentiated synovial sarcoma. Oncology was consulted with a plan of initiating chemotherapy and radiotherapy. However, the patient suffered a sudden cardiac arrest the following day, which was attributed to myocardial infarction. The cause of death could not be confirmed since an autopsy was not performed.

## Discussion

Primary pericardial synovial sarcoma is an exceedingly rare and aggressive tumor with a high risk of local invasion and poor prognosis. PPSS originates from pluripotent mesenchymal cells within the pericardium [5]. The characteristic molecular hallmark of synovial sarcoma is the chromosomal translocation t(X;18)(p11;q11), which causes the fusion of the SS18 or Synaptotagmin (SYT) gene on chromosome 18 with one of the SSX genes (SSX1, SSX2, or SSX4) on the X chromosome. The resultant SS18-SSX fusion protein functions as an oncogene, affecting the regulation of transcription and chromatin remodeling, leading to uncontrolled cellular proliferation and tumor development. The pericardium is highly vascular, which allows these synovial sarcomas with potential for fast local invasion. When seen macroscopically, PPSS manifests as a solid, lobulated, gray-white mass that is frequently attached to the pericardium. Histologically, PPSS is categorized mainly into two subtypes: monophasic and biphasic. However, if differentiation is very poor, there can be another entity called poorly differentiated. The monophasic type spindle cells (SS) are arranged in fascicles, and the cells are elongated with homogeneous nuclei and contain little cytoplasm. Necrosis and mitotic figures are frequently seen. On the other hand, biphasic SS consists of both spindle cell (mesenchymal) and epithelial components. In the spindle cell matrix, epithelial cells develop into glandular or papillary forms. Poorly differentiated SS consists of tiny, spherical, or polygonal cells with a high ratio of cytoplasm to nucleus. Necrosis, nuclear pleomorphism, and high mitotic activity are frequent [6].

Reverse transcription-polymerase chain reaction (RT-PCR) or fluorescence in situ hybridization (FISH) are the gold standards for diagnosing the SS18-SSX fusion gene; however, a number of additional markers can both confirm and rule out the diagnosis [7]. Immunohistochemical staining can aid in the diagnosis, help identify the subtype, and help eliminate other common tumors. Commonly utilized markers include Bcl-2, which exhibits substantial cytoplasmic positivity, and TLE1, which is very sensitive and specific for synovial sarcomas [8,9]. In biphasic subtypes, additional markers, including cytokeratins (AE1/AE3) and EMA, aid in identifying the epithelial components. In every subtype, vimentin, a mesenchymal marker, is consistently positive [10]. WT1 and calretinin are frequently negative, which helps to distinguish PPSS from mesothelioma [11]. Similarly, a negative CD31 and CD34 exclude angiosarcoma, which typically expresses these markers [12]. Desmin is also seldom expressed in PPSS, which helps distinguish it from rhabdomyosarcoma, where it is a common marker [13].

The diagnosis of primary pericardial synovial sarcoma can be challenging due to its rarity and nonspecific presentation. Multimodal imaging is crucial in characterizing pericardial malignancies and assessing their relation to adjacent structures. A chest radiograph is usually the first investigation and may demonstrate an enlarged cardiac silhouette. Echocardiography can identify pericardial effusion and mass effect, but it has limitations in defining the tumor's extent [14]. Advanced imaging, such as contrast-enhanced CT scans and cardiac MRIs, is the mainstay for the diagnosis and characterization of these tumors and helps delineate the tumor's size, location, and local invasion [15]. Although high-resolution 3T or 7T MRI provides better image resolution, these are not well-studied for cardiac tumors [16]. For chest pathologies, 1.5T MRI with contrast is adequate and is primarily used for assessing cardiac function and myocardial infiltration. The effect of a large pericardial tumor on cardiac kinetics can be easily assessed, with a greater field of view as compared to echocardiography. 18F-FDG PET/CT has long been known to predict overall survival, pathological response, and metastases in these cases and helps in the staging of this disease [17]. Although pericardial metastases are the most common pericardial tumors, primary pericardial tumors are usually large and solitary. Imaging features of these masses are usually nonspecific; for example, rare tumors like schwannomas of the pericardium demonstrate similar heterogenous enhancement with T2 hyperintensity as seen in other locations in the body, and a biopsy is usually needed for diagnosis [18].

There is no defined treatment strategy for PPSS since it is so uncommon. However, the course of treatment usually consists of a mix of chemotherapy, radiation therapy (RT), and surgery, much as with other synovial sarcomas. The cornerstone of PPSS treatment is surgical resection, intending to completely remove the tumor and obtain negative margins. FISH or RT-PCR is needed to confirm the diagnosis as it detects the presence of SS18-SSX fusion protein. The pericardium's position, the locally aggressive nature of the tumor, and closeness to vital tissues such as the heart, major arteries, and lungs make surgical excision difficult. When total resection is not possible, debulking surgery may be used to minimize tumor burden and relieve symptoms. Clear surgical margins are associated with an improved prognosis; however, total resection is generally difficult due to the tumor's infiltrative nature. In the majority of instances, tumor resection is followed by chemotherapy. The most often used chemotherapy regimen is doxorubicin-ifosfamide, which is effective in synovial sarcoma and other soft tissue sarcomas [19,20]. Radiation therapy (RT) is often used after surgery. RT has been proven to lower the incidence of local recurrence and enhance survival rates in synovial sarcoma patients. It is also considered in cases of inoperable tumors for symptomatic control, as it helps shrink tumor size and decrease symptoms of SOB or chest heaviness. Studies are still being conducted to generate a targeted therapy and immunotherapy against the SYT-SSX fusion protein, but these are still in experimental stages [21]. Similarly, clinical studies are currently undergoing to develop molecular drugs against the fusion gene. The prognosis for PPSS is typically poor due to the disease's nonspecific presentation, late diagnosis, and aggressive nature. The infiltrative growth pattern of PPSS complicates full surgical resection, and achieving clean surgical margins is a considerable difficulty. The tumor has a high recurrence rate and limited treatment therapies.

After reviewing the literature, we discovered 46 documented cases of PPSS, in addition to our report of two cases (Table 1).

**Table 1:** Primary pericardial synovial sarcoma cases This table outlines reported cases of primary pericardial sarcoma, including patient demographics, histological subtype, presenting symptoms, diagnostic modalities, treatment strategies (surgery, chemotherapy, or radiotherapy), and outcomes. M: male, F: female, SOB: shortness of breath, NA: not available, NI: not indicated, Neg: negative, CP: chest pain, CM: cardiomegaly, SVC: superior vena cava syndrome, HF: heart failure, SCD: sudden cardiac death, CT: chemotherapy, RT: radiotherapy, b/l: bilateral, IHC: immunohistochemistry

Year	Age	Ethnicity	Presenting symptom	Tumor size	Morphology	Genetic	Pericardial effusion	Effusion cytology	Local metastasis	Distant metastasis	Surgery	RT	CT	Outcome
1999 [22]	19/M	East Indian	SOB	10	Biphasic	t(X;18)	Yes	Neg	Yes	No	Yes	Yes	Yes	Alive at 12 months
1999 [23]	35/F	NA	CP	11	Monophasic	t(X;18)	NA	NA	NA	NA	NA	NA	NA	NA
1999 [24]	19/F	NA	SOB	11×14×7 cm	Biphasic	t(X;18)	Yes	Neg	No	NA	Yes	No	No	Died in 7 months
2003 [25]	29/M	North Indian	SOB	8.5×5×1 cm	Monophasic	Not done	No	NI	Yes	NA	Yes	Yes	Yes	Had local recurrence-unresectable at 13 months
2004 [26]	26/M	NA	SOB/dizziness	3.5×4.0 cm	Biphasic	t(X;18)	No	NI	No	No	Yes	Yes	Yes	Recurred 5 times, survived 14 years, but had pleural involvement; refused further treatment
2007 [27]	64/F	NA	SOB	NA	NA	Not done	Yes	No	NA	NA	Yes	NA	NA	NA
2009 [28]	49/M	NA	Chest pain/SOB	NA	Monophasic	Not done	Yes	Neg	Yes	Y-LN	Yes	Declined	Declined	NA
2009 [29]	70/F	NA	Cough and SOB	13×7×6 cm	NA	t(X;18)	No	NI	Yes	NA	Yes	Declined	Declined	Tumor doubled in size in 11.9 days; died in 79 days
2009 [30]	61/M	NA	SOB	6×4 cm	Biphasic	Not done	Yes	Neg	Yes	NA	Yes	No	Yes	Recurrence at 2 months
2011 [31]	19/M	NA	Cough, SOB, orthopnea	15×15×10 cm	Monophasic	Not done	Yes	Not done	Yes	No	Yes	No	Yes	NA
2011 [32]	54/F	NA	Chest pain/fever/malaise	10×10×11 cm	Biphasic	t(X;18)	Yes	Not done	No	No	Yes	Yes	Yes	Recurrence at 22 months with mediastinal metastases; put on palliative CT; died after 4 months
2012 [33]	31/M	NA	Fever, cough	10 cm	Biphasic	t(X;18)	Yes	Not done	NA	NA	Yes	Declined	Yes	Alive at 12 months
2012 [33]	31/M	NA	Fever, chest pain	8×8×2 cm	Monophasic	t(X;18)	Yes	Not done	Yes	No	Yes	Yes	Yes	Died 27 months after initial presentation
2012 [33]	38/M	NA	SOB	8 cm	Monophasic	t(X;18)	Yes	Not done	Yes	No	Yes	No	No	Was in remission for 2 weeks/awaiting transplant
2021 [34]	61/M	NA	SCD	5×3×2.5 cm	Biphasic	t(X;18)	NA	NA	NA	NA	NA	NA	NI	Discovered at autopsy
2013 [35]	13/M	NA	SOB	7.3×6.1 cm	Monophasic	t(X;18)	Yes	Not done	Yes	No	Yes	Yes	Yes	Relapsed in 21 months
2022 [36]	19/M	Chinese	Chest tightness	15.3×11.7×15.1 cm	Biphasic	t(X;18)	Yes	Neg	Yes	No	Yes	No	Yes	NA
2021 [37]	52/F	NA	Facial congestion/SVC syndrome	10×8×5 cm	Monophasic	Not done	Yes	Not done	No	No	Yes	No	Yes	Relapsed after 18 months, died 2 months after
2014 [38]	36/M	NA	Loss of appetite and fatigue	7×4.8 cm	Monophasic	t(X;18)	Y	Neg	Yes	No	Yes	No	Yes	NA
2014 [39]	27/M	Hispanic	Cough	5.2×1.3×0.7 cm	Monophasic	t(X;18)	Yes	Neg	No	No	Yes	Yes	Yes	Recurrence at 9 months
2015 [40]	57/M	NA	CM on X-ray	NA	NA	t(X;18)	Yes	Not done	Yes	No	Yes	Yes	Yes	Died after 3 years
2007 [41]	15/M	NA	Cough/orthopnea	NA	Monophasic	t(X;18)	NA	NA	NA	NA	Yes	No	Yes	Recurrence at 5 months; died at 31 months after initial diagnosis
2014 [42]	13/M	NA	Fever/chest pain	8 cm	Monophasic	t(X;18)	Yes	Neg	Yes	No	Yes	No	Yes	Alive at 3 months
2016 [43]	34/M	NA	Fever/chill, malaise	6.3×10.1 cm	Biphasic	t(X;18)	Yes	Neg	NA	NA	NA	NA	Na	Alive at 2 years
2010 [44]	51/M	NA	SOB	6.2×6.2×6 cm	Monophasic	t(X;18)	Yes	Neg	No	No	Yes	Yes	No	Alive at 9 months
2007 [45]	19/M	NA	SOB	10×10×15 cm	Monophasic	t(X;18)	Yes	Not done	Yes	Yes	No	No	No	Died at 3 months
2019 [46]	56/M	NA	SOB/fatigue	8.1×5.6 cm	Biphasic	NA	Yes	Not done	Yes	No	Yes	Yes	Yes	Alive at 36 months
2021 [47]	48/M	South Asian	Incidental b/l pleural effusions	13.5×5 cm	NA	t(X;18)	Yes	Neg	Yes	No	NA	NA	NA	NA
2009 [48]	38/F	Caucasian	SOB	15×10 cm size	Monophasic	t(X;18)	Yes	Not done	Yes	No	Yes	Yes	Yes	In remission at 22 months
2013 [49]	45/F	NA	SOB	3.8×5.2 cm	Biphasic	NA	Yes	Neg	No	No	Yes	No	Yes	Disease-free at 32 months
2009 [50]	41/F	NA	Cough/fever	NA	NA	NA	NA	NA	NA	NA	NA	NA	NA	NA
2023 [51]	35/F	Turkish	Back pain/palpitations	12×11×6.5 cm	Monophasic	t(X;18)	Yes	Spindle cell mesenchymal	No	No	Yes	Yes	Delayed, but yes	Died 21 months after initial resection
2024 [52]	52/M	NA	SOB	15×9×14 cm	NA	NA	No	NI	Yes	No	Yes		Yes	Alive at 15 months
2024 [53]	33/M	NA	SOB/CP	6×5.5×5 cm	NA	t(X;18)	Yes	Not done	NA	NA	Not done	No	Yes	Alive at 6 months
2024 [54]	35/F	NA	Fever/malaise	12.5×9×3 cm	Monophasic	t(X;18)	Yes	Not done	NA	NA	Yes	Yes	Yes	Alive at 1 year
2012 [55]	42/M	NA	SOB	8×4.8 cm	Monophasic	t(X;18)	Yes	Not done	Yes	No	Yes	Yes	Yes	Survives at 9 months after diagnosis
2023 [56]	34/M	NA	SOB	7.9×2.3 cm^2^	Monophasic	t(X;18)	Yes	Some spindle cells	Yes	No	Yes	No	Yes	Died at 17 months
2023 [57]	51/F	Latino	NA	NA	NA	NA	NA	NA	NA	NA	Yes	Yes	Yes	Remains asymptomatic for 5 years
2022 [58]	59/F	NA	Tamponade/HF	13 cm	NA	t(X;18)	Yes	Not done	NA	NA	Yes	No	Yes	Relapse at 3 months
2022 [59]	50/F	NA	Intermittent left chest pain	2.1×1.8×1.9 cm	NA	Not done, just based on IHC	No	NI	No	No	Yes	No	Yes	NA
2024 [60]	29/M	Fatigue, dyspnea	Fatigue, SOB	NA	NA	t(X;18)	Yes	Neg	Na	NA	Yes	Yes	Yes	Died within 1 month after diagnosis
2023 [61]	22/F	Likely Indian	SOB	25×20 cm	NA	Not done	Yes	Not done	Yes	No	Yes	No	Yes	NA
2020 [62]	42/M	Dyspnea	SOB	21×9×3 cm	Monophasic	t(X;18)	Yes	NA	NA	NA	NA	NA	NA	NA
2019 [63]	43/M	NA	Unstable angina	11×10×8 cm	Biphasic	t(X;18)	NA	NA	Yes	NA	Yes	No	No	Died 13 months after surgery
2024 [64]	64/F	NA	Chest tightness/SOB	8×4.5×6.7 cm	Monophasic	t(X;18)	No	NI	Yes	No	Yes	No	Yes	Died after 8 months
2004 [65]	22/F	NA	SOB	8×7 cm	Monophasic	t(X;18)	NA	NA	Yes	No	Yes	No	Yes	Alive at 12 months
2023	23/M	Caucasian	SOB	15.2×10.6 cm	Monophasic	NA	Present	Negative	Yes	No	Yes	No	Yes	Died 1 week after resection
2011	30/M	Caucasian	SOB, CP	16.7×14.5 cm	NA	NA	Neg	NA	Yes	No	Not done	No	Yes	Died after 11 months of presentation due to myocardial infarction

Of the 48 total cases, there were 17 female patients and 31 male patients. The ages of the patients ranged from 13 to 70 years. Ethnicity data were only available for 11 of these cases, including three Caucasians, four South Asians, one Turkish, one Latino, one Chinese, and one Hispanic. Twelve cases were classified as biphasic (i.e., the tumor had two different types of cells), and 23 cases were monophasic (i.e., the tumor had only one type of cell). For the remaining 13 cases, the subtype of the tumor was not specified. The most common symptom among these patients was shortness of breath (SOB), which was seen in 30 of the 48 (62%) cases. The second most common symptoms for presentation included fever, chest pain, and malaise (feeling unwell), which often looked like symptoms of viral pericarditis (inflammation of the lining around the heart), often leading to misdiagnosis. While most presenting symptoms are benign on presentation from our literature review, one patient was found dead on arrival due to sudden cardiac death, and two patients presented with unstable angina. Two patients, however, were asymptomatic but were found to have cardiomegaly and pleural effusions on routine examinations, prompting further investigation. We can conclude and highlight the importance that it can be asymptomatic and present with symptoms ranging from nonspecific SOB to unstable angina, and given the rarity of the tumor, diagnosing it can be challenging.

In our literature review of 48 cases, 34 (70%) presented with pericardial effusion, which explains why SOB was the most common presenting symptom, followed by pericarditis-like symptoms. Cytological analysis of the pericardial fluid was performed in 16 cases. Among these, 14 cases yielded negative results, and in two cases, spindle cells were identified. However, the presence of spindle cells was considered inconclusive for definitive diagnosis. This highlights that the cytology in pericardial sarcoma is a very poor diagnostic test. In seven cases, no pericardial effusion was detected. For another seven cases, the presence or absence of effusion could not be determined due to limited data availability. Thirty-three cases underwent advanced diagnostic procedures such as fluorescence in situ hybridization (FISH) or immunohistochemistry (IHC) to evaluate genetic or molecular markers. In 35 out of 48 cases, tumors had already spread out of the pericardial space at diagnosis; however, for 13 cases, information was not available. Of the reported 35 cases, disease spread had already occurred in 26 either to the myocardium or surrounding major arteries, while two cases had evidence of distant metastasis at diagnosis, including the diaphragm and renal arteries. It is important to emphasize that in two (4%) cases, patients were misdiagnosed, one being a teenager wrongly diagnosed as a case of thymoma and the other, a 59-year-old woman misdiagnosed as angiofibroma, again highlighting the rarity of pericardial synovial sarcoma and its distribution in age groups. One case was diagnosed post-mortem and confirmed through autopsy findings. Tumor size was reported for 41 out of 48 patients, with an average tumor size of 10.1 cm. The largest tumor measured 25 cm in the largest dimension. Surgical resection was performed in 38 of the 41 cases with known tumor size. Most had their diagnosis confirmed through histopathological examination after surgical resection.

Regarding the treatment, after initial debulking with surgery, chemotherapy and radiotherapy were given. Of the 48 patients, 21 did not receive radiotherapy, although the reasons were not specified. For eight patients, data regarding radiotherapy administration were unavailable. Nineteen patients were offered radiotherapy; however, three of them declined treatment for unspecified reasons. Of the 48 patients, chemotherapy status was unknown for six cases, and 31 underwent chemotherapy. Two patients, despite being eligible, declined treatment due to financial constraints. One patient received palliative chemotherapy, while another tragically passed away before a diagnosis could be established. Additionally, chemotherapy was delayed for one patient due to pregnancy, highlighting the complexities of managing cancer in special populations. Regarding outcomes, the longest recorded survival was 14 years in a patient who faced five recurrences, treated with chemotherapy and resection, ultimately transitioning to palliative care for the fifth case mentioned in the table. The average time to recurrence calculated from available data for nine patients was 11 months, with documented recurrence times ranging from as early as two months to as late as 22 months. Three patients with biphasic tumors had an average recurrence time of 12 months, while another five with monophasic tumors experienced recurrence at an average of 13 months. For one patient, the tumor subtype was not available.

## Conclusions

Pericardial synovial sarcoma is a rare malignancy that poses a substantial diagnostic difficulty due to its vague symptoms and clinical similarities to benign cardiac diseases. Patients may be asymptomatic or exhibit symptoms ranging from shortness of breath to potentially fatal occurrences such as unstable angina or abrupt cardiac death. The tumor can occur across a wide age range and is often misdiagnosed, particularly in younger individuals. Pericardial effusion is mostly present on initial imaging, but the cytology of pericardial fluid is usually non-diagnostic, although tumors are sitting inside the pericardial effusion. Surgical resection remains the primary method of diagnosis, as tissue biopsy and advanced diagnostic techniques, such as immunohistochemistry and FISH, are needed for diagnosis. The usual treatment is resection followed by chemotherapy and/or radiation. Individual patient circumstances, such as pregnancy and financial restraints, might make treatment options more difficult. The recurrence rate is high, regardless of the treatment modality used. The disease's aggressive character, as well as its ability to invade local areas very early, highlight the importance of clinical awareness and early intervention, which are essential for increasing diagnostic accuracy and patient outcomes.

## References

[1] Blay JY, von Mehren M, Jones RL (2023). Synovial sarcoma: characteristics, challenges, and evolving therapeutic strategies. ESMO Open.

[2] Yang K, Lui WO, Xie Y (2002). Co-existence of SYT-SSX1 and SYT-SSX2 fusions in synovial sarcomas. Oncogene.

[3] McAllister HA Jr, Hall RJ, Cooley DA (1999). Tumors of the heart and pericardium. Curr Probl Cardiol.

[4] Krieg AH, Hefti F, Speth BM (2011). Synovial sarcomas usually metastasize after >5 years: a multicenter retrospective analysis with minimum follow-up of 10 years for survivors. Ann Oncol.

[5] Restrepo CS, Vargas D, Ocazionez D, Martínez-Jiménez S, Betancourt Cuellar SL, Gutierrez FR (2013). Primary pericardial tumors. Radiographics.

[6] Ren C, Liu J, Hornicek FJ, Yue B, Duan Z (2024). Advances of SS18-SSX fusion gene in synovial sarcoma: emerging novel functions and therapeutic potentials. Biochim Biophys Acta Rev Cancer.

[7] Lasota J, Chłopek M, Kaczorowski M (2024). Utility of immunohistochemistry with antibodies to SS18-SSX chimeric proteins and C-terminus of SSX protein for synovial sarcoma differential diagnosis. Am J Surg Pathol.

[8] Jagdis A, Rubin BP, Tubbs RR, Pacheco M, Nielsen TO (2009). Prospective evaluation of TLE1 as a diagnostic immunohistochemical marker in synovial sarcoma. Am J Surg Pathol.

[9] Rekhi B, Basak R, Desai SB, Jambhekar NA (2012). Immunohistochemical validation of TLE1, a novel marker, for synovial sarcomas. Indian J Med Res.

[10] Pelmus M, Guillou L, Hostein I, Sierankowski G, Lussan C, Coindre JM (2002). Monophasic fibrous and poorly differentiated synovial sarcoma: immunohistochemical reassessment of 60 t(X;18)(SYT-SSX)-positive cases. Am J Surg Pathol.

[11] Miettinen M, Limon J, Niezabitowski A, Lasota J (2001). Calretinin and other mesothelioma markers in synovial sarcoma: analysis of antigenic similarities and differences with malignant mesothelioma. Am J Surg Pathol.

[12] Rao P, Lahat G, Arnold C (2013). Angiosarcoma: a tissue microarray study with diagnostic implications. Am J Dermatopathol.

[13] Parham DM, Webber B, Holt H, Williams WK, Maurer H (1991). Immunohistochemical study of childhood rhabdomyosarcomas and related neoplasms. Results of an intergroup rhabdomyosarcoma study project. Cancer.

[14] Goldblatt J, Saxena P, McGiffin DC, Zimmet A (2015). Pericardial synovial sarcoma: a rare clinical entity. J Card Surg.

[15] Assunção FB, de Oliveira DC, Souza VF, Nacif MS (2016). Cardiac magnetic resonance imaging and computed tomography in ischemic cardiomyopathy: an update. Radiol Bras.

[16] Kumar T, Virador GM, Brahmbhatt P (2023). High-resolution 7T MR imaging of the trochlear nerve. AJNR Am J Neuroradiol.

[17] Chang KJ, Lim I, Park JY (2015). The role of (18)F-FDG PET/CT as a prognostic factor in patients with synovial sarcoma. Nucl Med Mol Imaging.

[18] Brahmbhatt P, Kumar T, Bhatt AA (2023). Sinonasal schwannomas: imaging findings and review of literature. Ear Nose Throat J.

[19] von Mehren M, Kane JM, Agulnik M (2022). Soft tissue sarcoma, version 2.2022, NCCN Clinical Practice Guidelines in Oncology. J Natl Compr Canc Netw.

[20] Judson I, Verweij J, Gelderblom H (2014). Doxorubicin alone versus intensified doxorubicin plus ifosfamide for first-line treatment of advanced or metastatic soft-tissue sarcoma: a randomised controlled phase 3 trial. Lancet Oncol.

[21] Fuchs JW, Schulte BC, Fuchs JR, Agulnik M (2023). Targeted therapies for the treatment of soft tissue sarcoma. Front Oncol.

[22] Al-Rajhi N, Husain S, Coupland R, McNamee C, Jha N (1999). Primary pericardial synovial sarcoma: a case report and literature review. J Surg Oncol.

[23] Kojima KY, Koslin DB, Primack SL, Kettler MD (1999). Synovial sarcoma arising from the pericardium: radiographic and CT findings. AJR Am J Roentgenol.

[24] Oizumi S, Igarashi K, Takenaka T, Yamashiro K, Hiraga H, Fujino T, Horimoto M (1999). Primary pericardial synovial sarcoma with detection of the chimeric transcript SYT-SSX. Jpn Circ J.

[25] Anand AK, Khanna A, Sinha SK, Mukherjee U, Walia JS, Singh AN (2003). Pericardial synovial sarcoma. Clin Oncol (R Coll Radiol).

[26] Van der Mieren G, Willems S, Sciot R, Dumez H, Van Oosterom A, Flameng W, Herijgers P (2004). Pericardial synovial sarcoma: 14-year survival with multimodality therapy. Ann Thorac Surg.

[27] Schumann C, Kunze M, Kochs M, Hombach V, Rasche V (2007). Pericardial synovial sarcoma mimicking pericarditis in findings of cardiac magnetic resonance imaging. Int J Cardiol.

[28] Korula A, Shah A, Philip MA, Kuruvila K, Pradhip J, Pai MC, Chacko RT (2009). Primary mediastinal synovial sarcoma with transdiaphragmatic extension presenting as a pericardial effusion. Singapore Med J.

[29] Katakura H, Fukuse T, Shiraishi I (2009). Mediastinal synovial sarcoma. Thorac Cardiovasc Surg.

[30] Moorjani N, Peebles C, Gallagher P, Tsang G (2009). Pericardial synovial sarcoma. J Card Surg.

[31] Imran R, Ahmad BM, Aziz SA, Salma B, Charoo ML (2011). Primary synovial sarcoma of pericardium: a case report. Chin Ger J Clin Oncol.

[32] Akerström F, Santos B, Alguacil AM, Orradre JL, Lima P, Zapardiel S (2011). Pericardial synovial sarcoma. Thorac Cardiovasc Surg.

[33] Cheng Y, Sheng W, Zhou X, Wang J (2012). Pericardial synovial sarcoma, a potential for misdiagnosis: clinicopathologic and molecular cytogenetic analysis of three cases with literature review. Am J Clin Pathol.

[34] Kodikara S (2012). Pericardium: an exceedingly rare site for a primary biphasic synovial sarcoma. Indian J Pathol Microbiol.

[35] Chekrine T, Sahraoui S, Cherkaoui S (2014). Primary pericardial synovial sarcoma: a case report and literature review. J Cardiol Cases.

[36] Luo Y, Gong K, Xie T (2022). Case report: a young man with giant pericardial synovial sarcoma. Front Cardiovasc Med.

[37] Manole S, Pintican R, Palade E (2022). Primary pericardial synovial sarcoma: a case report and literature review. Diagnostics (Basel).

[38] Ohzeki M, Fujita S, Miyazaki H (2014). A patient with primary pericardial synovial sarcoma who presented with cardiac tamponade: a case report and review of the literature. Intern Med.

[39] Phatak P, Khanagavi J, Aronow WS, Puri S, Yusuf Y, Puccio C (2014). Pericardial synovial sarcoma: challenges in diagnosis and management. F1000Res.

[40] Muramatsu T, Takeshita S, Tanaka Y, Morooka H, Higure R, Shiono M (2015). Primary pericardial synovial sarcoma. J Thorac Dis.

[41] Hing SN, Marshall L, Al-Saadi R, Hargrave D (2007). Primary pericardial synovial sarcoma confirmed by molecular genetic studies: a case report. J Pediatr Hematol Oncol.

[42] Yoshino M, Sekine Y, Koh E (2014). Pericardial synovial sarcoma: a case report and review of the literature. Surg Today.

[43] Youn HC, Lee Y, Kim SC (2016). Pericardial synovial sarcoma presenting with large recurrent pericardial effusion. J Thorac Dis.

[44] Yokouchi Y, Hiruta N, Oharaseki T (2011). Primary cardiac synovial sarcoma: a case report and literature review. Pathol Int.

[45] de Zwaan C, Bekkers SC, van Garsse LA, Jansen RL, van Suylen RJ (2007). Primary monophasic mediastinal, cardiac and pericardial synovial sarcoma: a young man in distress. Neth Heart J.

[46] Duran-Moreno J, Kampoli K, Kapetanakis EI (2019). Pericardial synovial sarcoma: case report, literature review and pooled analysis. In Vivo.

[47] Chapra AF, Maliyakkal AM, Naushad VA, Valiyakath HS, Ahmed MS (2021). Primary pericardial synovial sarcoma: an extremely rare cardiac neoplasm. Cureus.

[48] Talukder M, Joyce L, Marks R, Kaplan K (2010). Primary cardiac synovial sarcoma. Interact Cardiovasc Thorac Surg.

[49] Wu X, Chen R, Zhao B (2013). Pericardial synovial sarcoma in a dyspnoeic female with tuberculous pericarditis: a case report. Oncol Lett.

[50] Romero-Castro R, Rios-Martin JJ, Gallego-Garcia de Vinuesa P (2009). Pericardial tumor diagnosed by EUS-guided FNA (with video). Gastrointest Endosc.

[51] Yaprak Bayrak B, Vural C, Sezer HF, Eliçora A, Busra Y (2023). Monophasic pericardial synovial sarcoma in a turkish female patient: a very rare case with cyto-histopathological findings. J Cardiothorac Surg.

[52] Ravishankar R, Makam R, Loubani M, Chaudhry M, Hussain A (2024). Primary pericardial synovial sarcoma requiring emergency salvage right atrial debulking: a case report. J Surg Case Rep.

[53] Haider J, Mahmood H, Faheem M, Khurshid S (2024). Pericardial synovial sarcoma in a young adult: case report of a rare malignancy. Ecancermedicalscience.

[54] Kawasaki T, Nakajima T, Torigoe T (2024). Case report: characteristics and nature of primary cardiac synovial sarcoma. Front Oncol.

[55] Ren Q, Saba SG, Heo S (2012). Primary pericardial synovial sarcoma: a rare case report with FISH analysis and review of literature. Am J Clin Pathol.

[56] Jin H, Zhang Y, Zhang W, Wang K (2023). Multimodal imaging features of primary pericardial synovial sarcoma: a case report. Front Oncol.

[57] Cianciulli TF, Saccheri MC, Lax JA (2023). [Primary pericardial synovial sarcoma]. Medicina (B Aires).

[58] Kanemitsu Y, Inoue N, Sirakawa A, Mandai K, Kawamura T, Uno K (2022). Mediastinal primary synovial sarcoma with postoperative pericardial recurrence in a previously treated angiofibroma. Cureus.

[59] Rahmaniar D, Maranatha D (2022). The early-stage of primary pulmonary synovial sarcoma: a case report. Int J Surg Case Rep.

[60] Ravindran A, Miller MS, Ayers E, Gimple L, Ayers M (2024). A case report of primary pericardial sarcoma. Eur Heart J Case Rep.

[61] Kumar S, Rahul K, Tewarson V, Kumar B, Hakim MZ, Kumar S, Singh SK (2023). Cardiac synovial sarcoma masquerading as effusive constrictive pericarditis. Indian J Thorac Cardiovasc Surg.

[62] Saba SG, Ren Q, Perle MA (2021). An unusual cause of cardiac tamponade: primary pericardial synovial sarcoma. J Cardiovasc Imaging.

[63] Wong KY, Hong E, Fong CM, Wong PS (2020). Pericardial synovial sarcoma presenting with unstable angina. Asian Cardiovasc Thorac Ann.

[64] Xu N, Xie K, Xin D, Liang Z, Zeng Y (2024). Primary cardiac synovial sarcoma originating from the atrial septum and associated pulmonary infarction: a case report. J Cancer Res Clin Oncol.

[65] Yano M, Toyooka S, Tsukuda K (2004). SYT-SSX fusion genes in synovial sarcoma of the thorax. Lung Cancer.

